# Supplementary material for: Olfaction in fruit flies (Tephritidae) balances detection and discrimination of host fruits
Source: Commun Biol. 2026 Mar 3;9:512. doi: 10.1038/s42003-026-09751-3 (PMC13066201; doi:10.1038/s42003-026-09751-3)
Supplement: Supplementary file 2 — Supplemental Information [file 42003_2026_9751_MOESM2_ESM.pdf]

Supplementary Table. Synthetic compounds used in electrophysiological and behavioural assays. For each compound, the retention index (RI) measured in intact and sliced fruits samples is provided, along twith the RI of the corresponding synthetic standard. NIST RI values are reported as mean ± variation (number of sources). RI values in plant samples that differed by more than 10 units from the corresponding synthetic standard are shown in bold and marked with an asterisk (*). When this occurred, the compound identity was considered unconfirmed in that sample and excluded from further analyses. Compounds were asigned to two distinct blends for EAD assays (A and B) and for behavioural assays (species-specific and fruit-shared). Inclusion of a compound in the EAG_3 assay is indicated by an "x".

CAS	compound	RI intact fruits	RI sliced fruits	RI standards	NIST RI	EAG_3	GC-EAD_3 blend #	behavioural assay blend #	sharednes s$_{IF}$[28]	sharednes s$_{SF}$[28]
127-91-3	(-)-β-pinene	991	988	980	979±2 (849)	x	B		2.659	3.006
5794-03-6	(+)-camphene	959	956	951	951±3 (2)		A		1.735	0.684
3016-19-1	(4E,6E)-allocimene	1144	1144	1143	1144±1 (4)	x	B		1.498	1.827
53398-83-7	(E)-2-hexenyl butyrate	1192	1192	1195	1195±1 (8)	x	A		1.340	1.981
3779-61-1	(E)-β-Ocimene	1049	1051	1049	1049±2 (548)		A		1.748	2.173
3338-55-4	(Z)-β-Ocimene	1037	1037	1038	1038±2 (446)	x	A		1.748	2.173
13466-78-9	3-carene	1016	1013	1014	1011±2 (336)	x	B		2.042	2.229
140-11-4	benzyl acetate	1165	1166	1166	1164±2 (64)		A		0.500	1.277
103-37-7	benzyl butanoate	1351	1352	1349	1345±2 (17)		A		0.000	0.662
103-28-6	benzyl isobutyrate	1301	1300	1300	1305±1 (5)	x	B		0.000	0.000
109-21-7	butyl butyrate	994	993	995	995±2 (38)	x		species-specific	0.530	1.040
3681-71-8	cis-3-hexenyl acetate	1004	1003	1007	1005±2 (74)	x	B	fruit-shared	2.198	2.640
141-78-6	ethyl acetate	608	606	607	612±5 (113)	x		fruit-shared	2.659	2.558
105-54-4	ethyl butyrate	797	797	801	802±2 (154)	x	A	fruit-shared	1.611	2.427
106-30-9	ethyl heptanoate	1095	1095	1098	1097±3 (37)	x	A		0.562	0.802
123-66-0	ethyl hexanoate	997	998	998	1000±2 (159)	x	B		1.431	2.092
108-64-5	ethyl isovalerate	850	850	851	854±2 (71)	x	B		0.562	1.326
97-63-2	ethyl methacrylate	-	**812***	783	814±N/A (1)		B		-	0.685
105-37-3	ethyl propionate	707	-	710	709±4 (63)	x	A	fruit-shared	2.027	2.181
142-92-7	hexyl acetate	1009	1011	1013	1011±4 (112)		A	fruit-shared	2.568	1.488
110-19-0	isobutyl acetate	768	769	770	771±6 (43)	x	B		-	2.005
138-86-3	limonene	1038	1039	1032	1030±2 (1005)	x	B		2.957	2.791
78-70-6	linalool	1101	1102	1102	1099±2 (976)	x	B		0.500	2.378

868-57-5	methyl 2-methylbutyrate	772	772	775	774±3 (48)	x	A	species-specific	0.849	1.509
623-42-7	methyl butyrate	718	717	719	722±3 (59)	x	A		0.000	2.118
106-73-0	methyl heptanoate	1023	1022	1025	1023±3 (16)		A	species-specific	0.562	0.562
106-70-7	methyl hexanoate	928	923	924	925±3 (69)	x	A		1.093	1.901
111-11-5	methyl octanoate	1122	1122	1125	1126±2 (51)		A	species-specific	0.931	1.657
119-36-8	methyl salicylate	1203	1203	1197	1192±2 (145)	x	B		1.418	2.058
624-24-8	methyl valerate	832	831	822	823±2 (17)	x	A		0.000	1.282
123-35-3	myrcene	989	990	992	991±2 (841)	x	A		0.690	2.647
628-63-7	pentyl acetate	-	910	913	911±6 (40)	x	A		-	1.168
122-72-5	phenylpropyl acetate	1376	1377	1374	1373±6 (14)		B		0.000	0.637
1191-16-8	prenyl acetate	919	915	922	918±4 (11)	x	B	species-specific	0.000	1.791
109-60-4	propyl acetate	711	709	712	708±8 (55)	x	B		-	1.933
106-36-5	propyl propionate	804	806	808	807±6 (17)	x	B		0.000	0.000
80-56-8	α-pinene	941	941	936	937±3 (996)	x	B		2.808	2.821
98-55-5	α-terpineol	1204	1204	1194	1189±2 (811)	x			1.761	1.730
87-44-5	β-caryophyllene	**1447***	1427	1428	1419±3 (983)	x	B		1.386	1.349

9

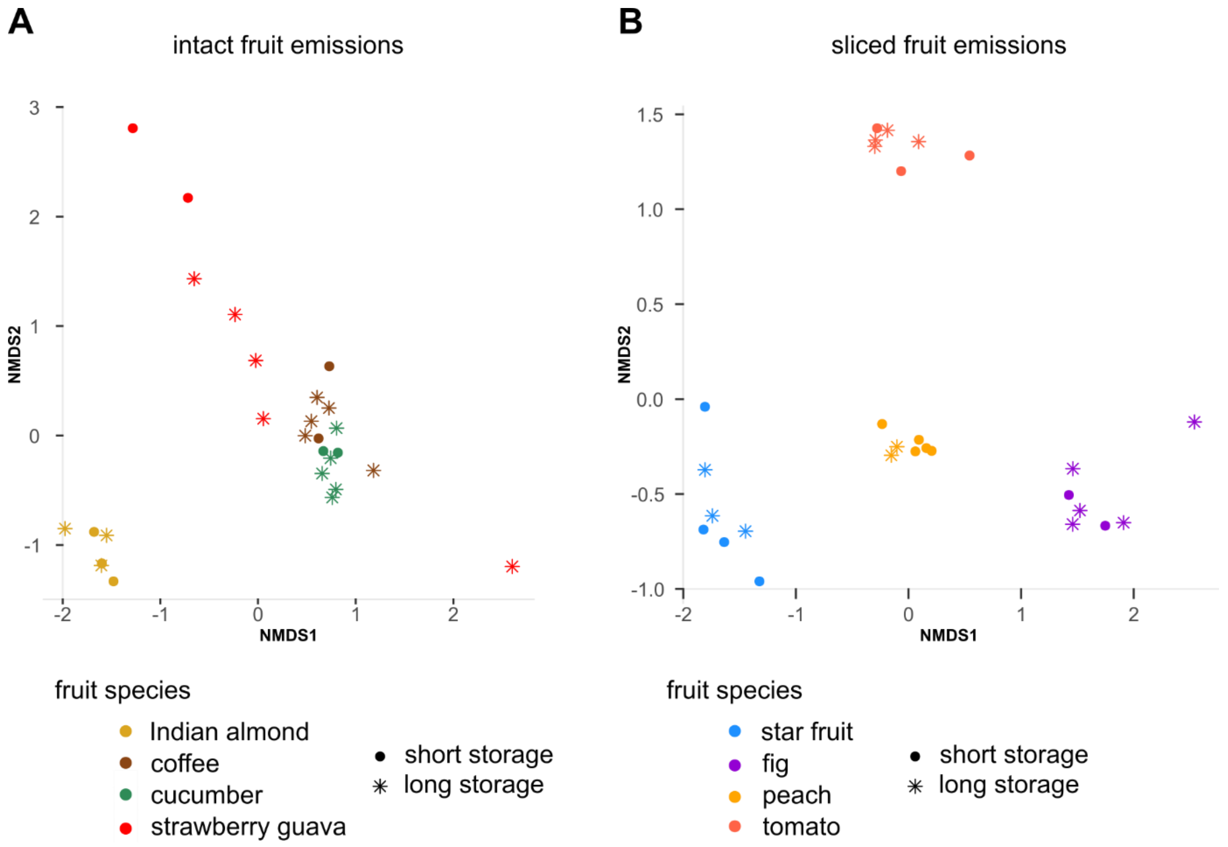

Supplementary Figure 1. Multivariate analysis of fruit emission samples stored in Tenax cartridges for both short and long time periods. (A) For intact fruit emissions, the long storage condition exceeded the short storage condition by three days for Indian almond, three to four days for coffee, four to five days for cucumbers, and six days for strawberry guava. (B) For sliced fruit, the additional storage time between the two conditions was six days for star fruit, three to four days for fig, four days for peach, and seven days for tomato. Possible degradation of strawberry guava samples during storage may have occurred, but was negligible for the other fruits. No statistics are given due to the low number of samples.

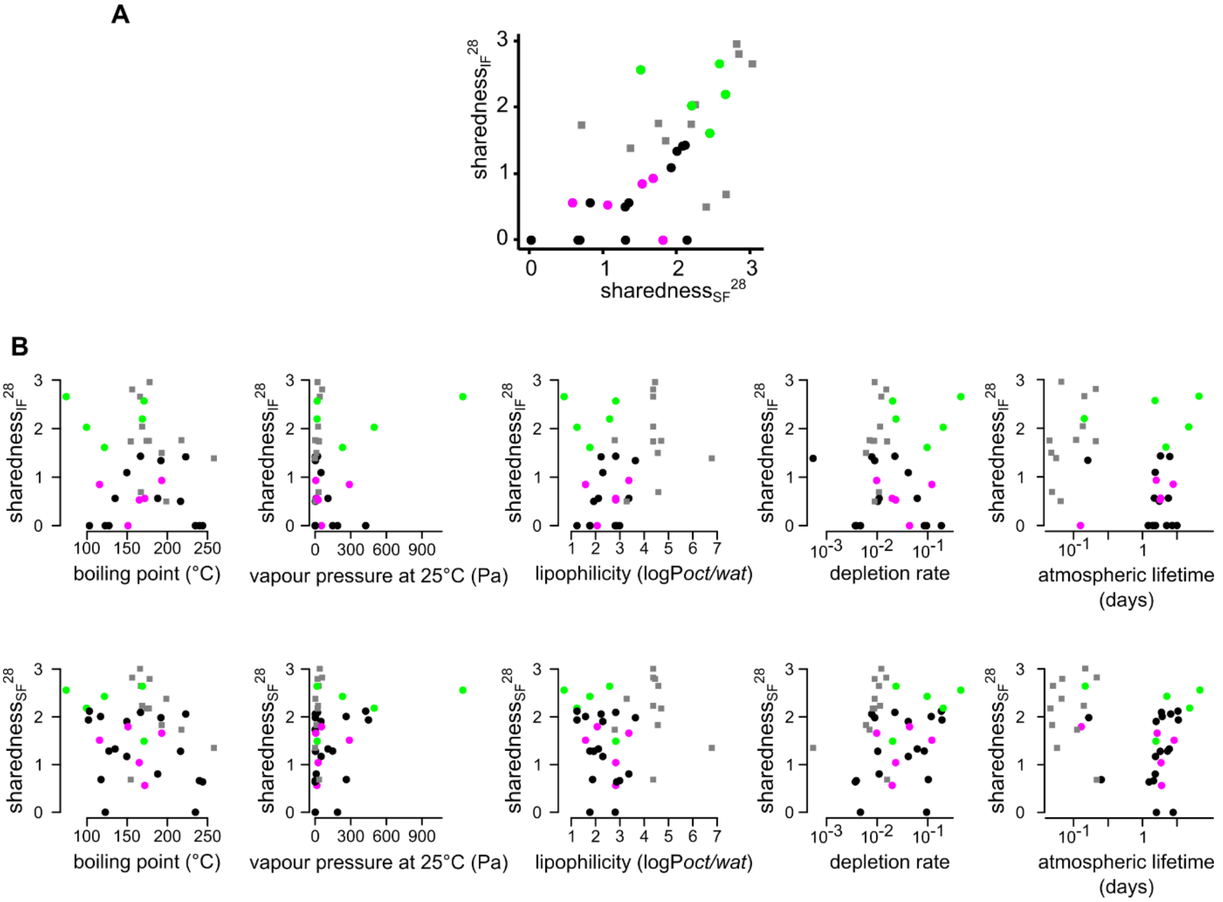

Supplementary Figure 2. Chemical characteristics of the compounds used for testing the olfactory system. (A) Relationship between the fruit sharedness indices calculated from intact and sliced fruit. Coloured dots show the compounds used for behavioural tests: the five compounds included in the species-specific compound blend are in magenta and the five compounds included in the shared fruit compound blend are in green. Terpenoids are shown by grey squares. (B) Indices of sharedness in functions of several chemical properties. Same conventions as in panel (A).

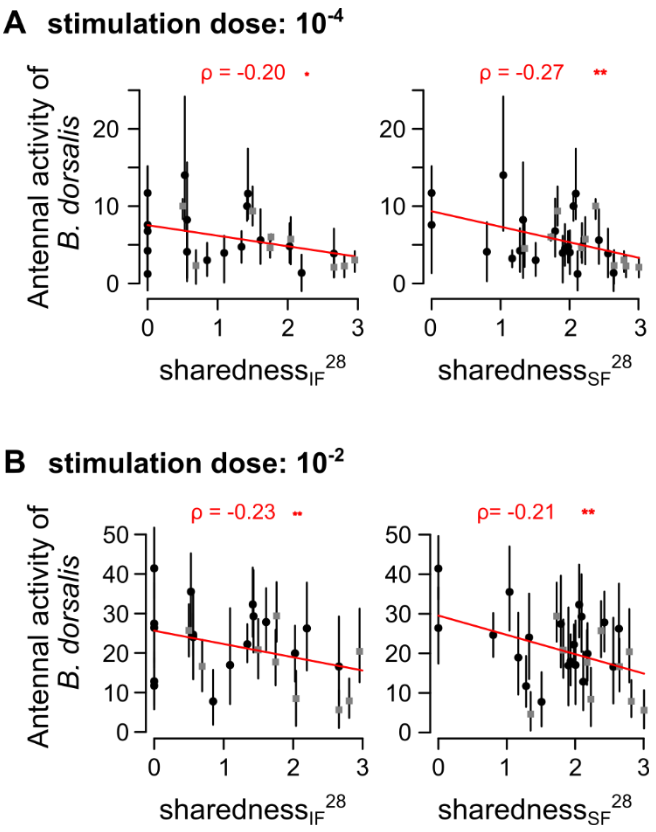

Supplementary Figure 3. Antennal responses of *Bactrocera dorsalis* measured with EAG_3 correlate with sharedness indices calculated from intact and sliced fruit emissions. (A-B) Scatterplot at the two doses tested. Same conventions as in Figure 3.

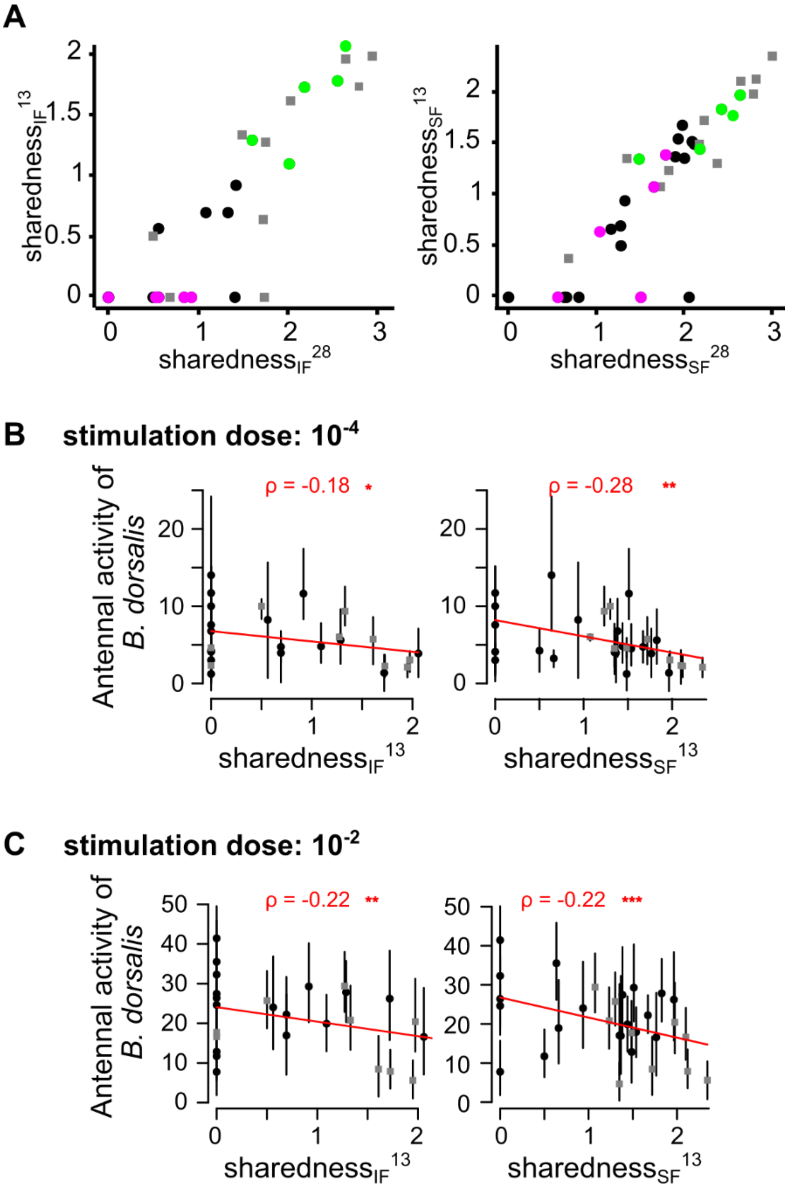

Supplementary Figure 4. Antennal responses of *Bactrocera dorsalis* measured with EAG_3 correlate with indices of sharedness. Indices of sharedness were calculated from a subset of 13 intact or sliced fruit emissions (Table 1), at the two doses tested. (A) relationship between sharedness indices calculated from 13 of the 28 fruit species. (B-C) Scatterplots at the two dose tested. Same conventions as in Figure S2 and S3.

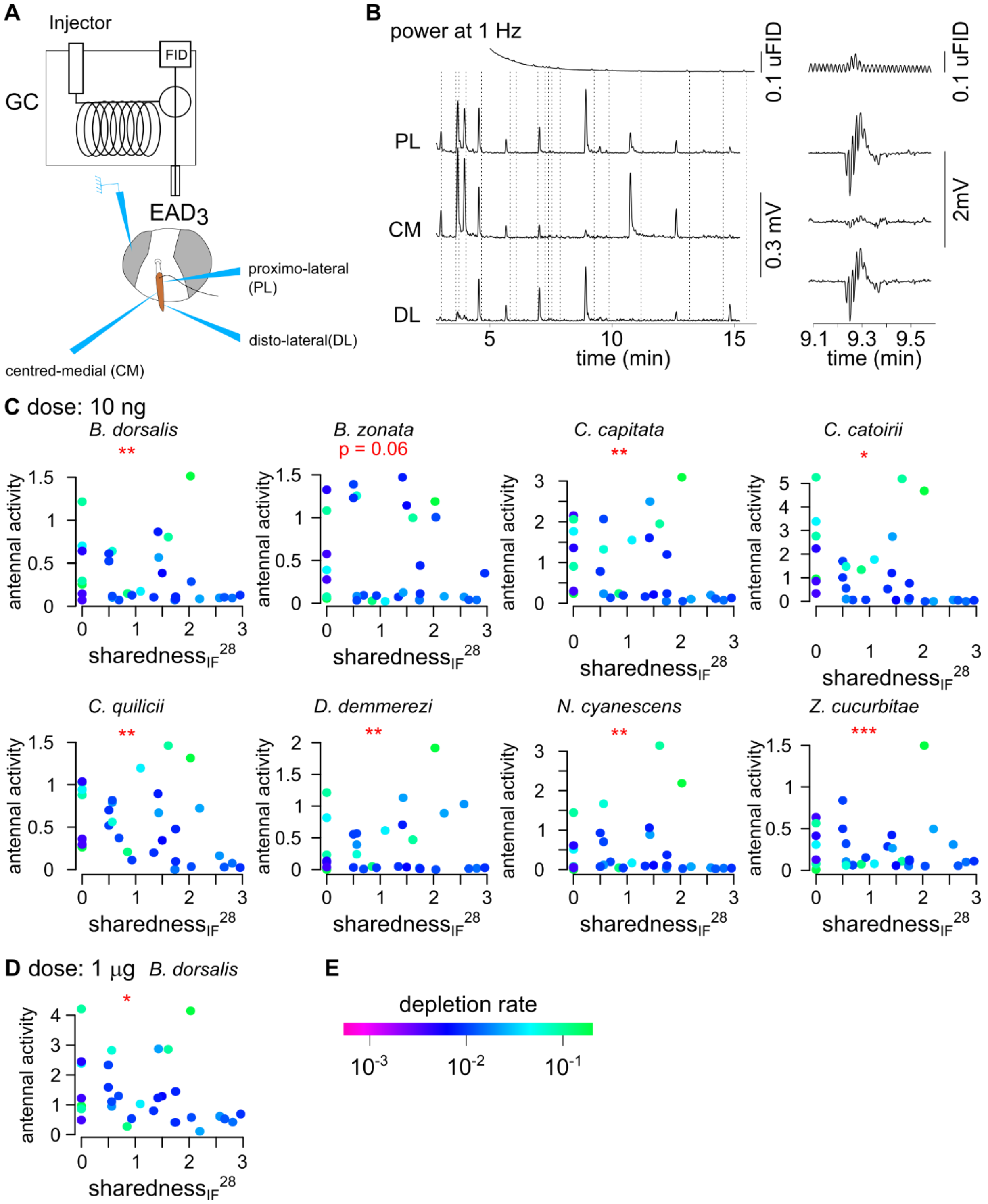
A
Injector
FID
GC
EAD3
proximo-lateral (PL)
disto-lateral(DL)
centred-medial (CM)
B
power at 1 Hz
PL
CM
DL
0.1 uFID
0.3 mV
2mV
time (min)
C dose: 10 ng
B. dorsalis
B. zonata
p = 0.06
C. capitata
C. catoirii
C. quilicii
D. demmerezi
N. cyanescens
Z. cucurbitae
antennal activity
sharedness$_{IF}^{28}$
D dose: 1 µg B. dorsalis
E
depletion rate
10^{-3}
10^{-2}
10^{-1}

Supplementary Figure 5. Antennal responses of eight Tephritidae species in GC-EAD$_3$. (A) Schematic representation of the chopped GC-EAD$_3$ system. (B) An example of GC-EAD$_3$ recording on individual female *B. dorsalis*. Left: Signal is demodulated at 1 Hz, the frequency of the chopping modulation. The first line is the FID signal and the three lines below are the EAD signals at the three antennal positions. Right: zoom on a single response, showing the full signal before demodulation. (C-D) Antennal responses to synthetic compounds at dose 10 ng (C) and 1 µg (D, n=4), depending on the degree of sharedness among intact fruit emissions. Each point represents a mean response to a compound (n = 4 for dose 10 ng, n = 5 for dose 1 µg). (E) Compound depletion rate is colour coded. The significance of a correlation between antennal activity and an interaction between sharedness$_{IF}$[28] and depletion rate is indicated (* $p < 0.05$, ** $p < 0.01$, *** $p < 0.001$).

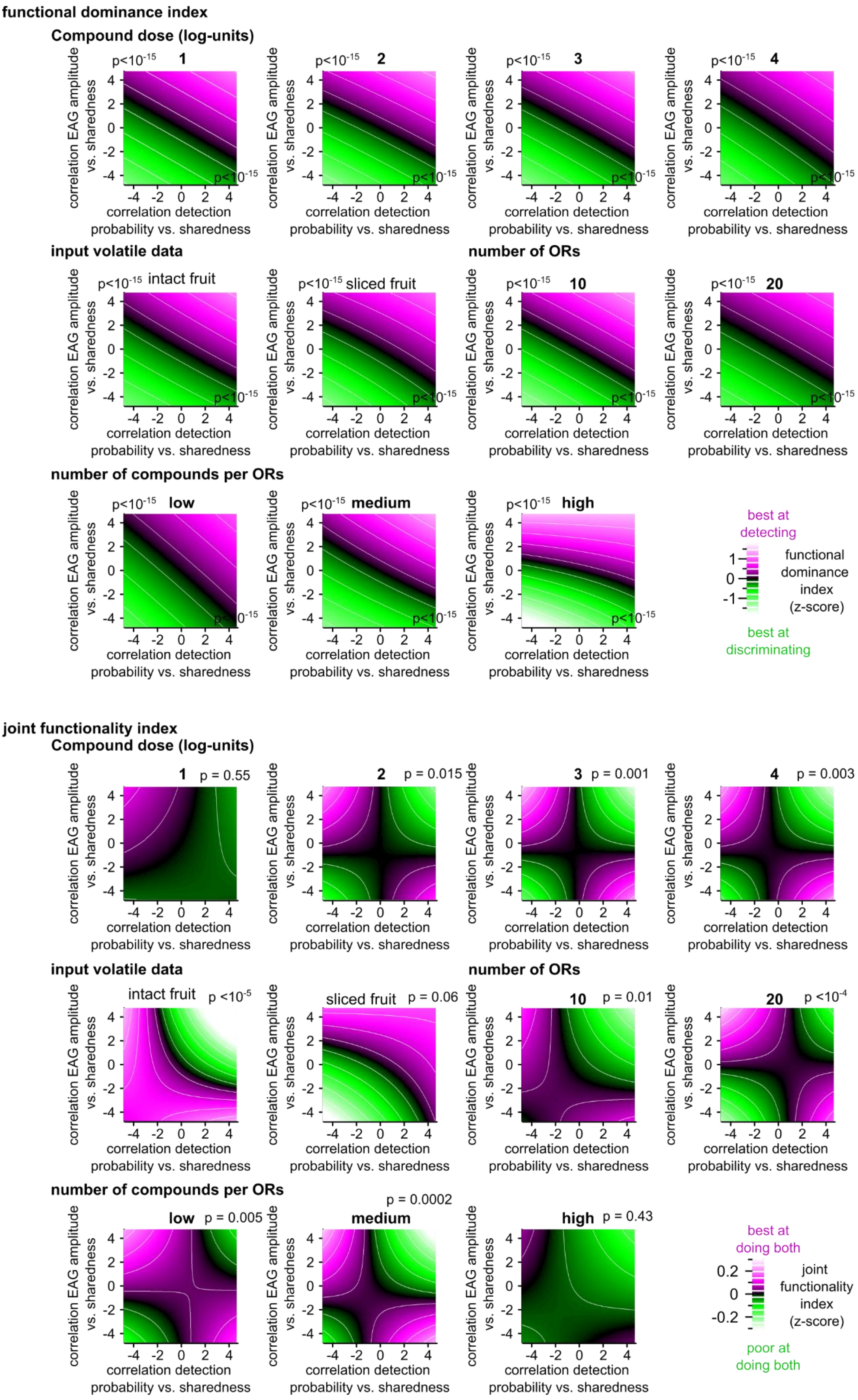
functional dominance index
Compound dose (log-units)
1
2
3
4
p<10-15
correlation EAG amplitude vs. sharedness
correlation detection probability vs. sharedness
input volatile data
intact fruit
sliced fruit
number of ORs
10
20
number of compounds per ORs
low
medium
high
best at detecting
functional dominance index (z-score)
best at discriminating
joint functionality index
p = 0.55
p = 0.015
p = 0.001
p = 0.003
p <10-5
p = 0.06
p = 0.01
p <10-4
p = 0.005
p = 0.0002
p = 0.43
best at doing both
joint functionality index (z-score)
poor at doing both

51

Supplementary Figure 6. Functional dominance index (top graphs) and fruit detectability index (bottom graphs) dependence on EAG response properties, as in Figure 4, depicted for all stimulation dose, input volatile data, number of ORs in the model, and number of compounds per ORs in the model. The functional dominance index is simply the difference between detectability and discriminability indices. The p-values indicate how much it depends on the two axis in each case (linear model). For joint functionality index, the p-values indicate how much it depends on an interaction between the two axis.

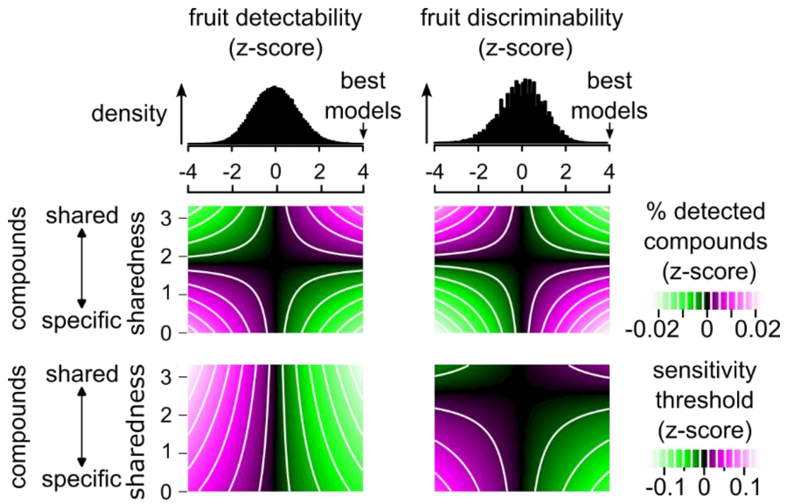

Supplementary Figure 7. Model parameters for 120,000 models with random connectivity. Top: the density distribution of fruit detectability and the fruit-species discriminability index for the 120,000 models. The best models for the two functions are on the upper part of the distribution. The linear projection of the proportion of compounds detected by the olfactory system (upper heatmaps) and that of the sensitivity threshold of detected compounds (lower heatmaps) are drawn on the spaces delineated by the model's fruit detectability indices (left heatmaps) and fruit-species discriminability indices (right heatmaps), as the x-axis, and compound sharedness as the y-axis. that response parameters. The proportion of detected compounds, the sensitivity thresholds of detected compounds and, to a lesser extent, the dynamic range of detected compounds depended on a joint effect between the compound's sharedness index and the fruit detectability index ($F (1, df > 10^7) = 285$; $p < 10^{-15}$; $F (1, df > 10^7) = 613$; $p < 10^{-15}$; and $F (1, df > 10^7) = 34.3$; $p < 10^{-8}$, respectively). Specifically, olfactory systems that are efficient at detecting fruit perceive shared compounds better than species-specific ones. Inversely, the same response parameters depended on a joint effect between sharedness index and fruit species discriminability index, namely the proportion of detected compounds (F (1, df > 107) = 453; p < 10-15), the sensitivity thresholds of detected compounds (F (1, df > 107) = 731; p < 10-15) and, to a lesser extent, the dynamic range of detected compounds (F (1, df > 107) = 18.6; p < 10-4). Olfactory systems capable of discriminating between fruit species efficiently are better at detecting species-specific compounds than shared compounds.
