# Supplementary material for: Olfaction in fruit flies (Tephritidae) balances detection and discrimination of host fruits
Source: Commun Biol. 2026 Mar 3;9:512. doi: 10.1038/s42003-026-09751-3 (PMC13066201; doi:10.1038/s42003-026-09751-3)
Supplement: Supplementary file 3 — Description of Additional Supplementary Materials [file 42003_2026_9751_MOESM3_ESM.pdf]

Description of Additional Supplementary Files

File name: Supplementary Data 1

Description: List of tentative names, classes, CAS number, sharedness^{28} , sharedness^{13} , Cmean and atmospheric lifetime values for the 511 compounds from intact fruit samples.

File name: Supplementary Data 2

Description: List of tentative names, classes, CAS number, sharedness^{28} , sharedness^{13} , Cmean and atmospheric lifetime values for the 665 compounds from sliced fruit samples.
