# Supplementary material for: Olfaction in fruit flies (Tephritidae) balances detection and discrimination of host fruits
Source: Commun Biol. 2026 Mar 3;9:512. doi: 10.1038/s42003-026-09751-3 (PMC13066201; doi:10.1038/s42003-026-09751-3)
Supplement: Supplementary file 6 — Reporting Summary [file 42003_2026_9751_MOESM6_ESM.pdf]

nature portfolio

Corresponding author(s): Vincent Jacob

Last updated by author(s): Oct 29, 2024

Reporting Summary

Nature Portfolio wishes to improve the reproducibility of the work that we publish. This form provides structure for consistency and transparency in reporting. For further information on Nature Portfolio policies, see our Editorial Policies and the Editorial Policy Checklist.

Statistics

For all statistical analyses, confirm that the following items are present in the figure legend, table legend, main text, or Methods section.

n/a	Confirmed	
☐	☒	The exact sample size (*n*) for each experimental group/condition, given as a discrete number and unit of measurement
☐	☒	A statement on whether measurements were taken from distinct samples or whether the same sample was measured repeatedly
☐	☒	The statistical test(s) used AND whether they are one- or two-sided<br>*Only common tests should be described solely by name; describe more complex techniques in the Methods section.*
☐	☒	A description of all covariates tested
☒	☐	A description of any assumptions or corrections, such as tests of normality and adjustment for multiple comparisons
☐	☒	A full description of the statistical parameters including central tendency (e.g. means) or other basic estimates (e.g. regression coefficient) AND variation (e.g. standard deviation) or associated estimates of uncertainty (e.g. confidence intervals)
☐	☒	For null hypothesis testing, the test statistic (e.g. *F*, *t*, *r*) with confidence intervals, effect sizes, degrees of freedom and *P* value noted<br>*Give P values as exact values whenever suitable.*
☒	☐	For Bayesian analysis, information on the choice of priors and Markov chain Monte Carlo settings
☒	☐	For hierarchical and complex designs, identification of the appropriate level for tests and full reporting of outcomes
☐	☒	Estimates of effect sizes (e.g. Cohen's *d*, Pearson's *r*), indicating how they were calculated

Our web collection on statistics for biologists contains articles on many of the points above.

Software and code

Policy information about availability of computer code

Data collection	We used the softwares Labview for collecting electrophysiological data, and TurboMass for collecting GC-MS data
Data analysis	We used the openSource software R, and MzMine2 for chemical analysis.<br>Codes were deposited in a long-term repository, will be made available publicly upon publication, and are available for reviewers and editors on the following private link:<br>https://dataverse.cirad.fr/privateurl.xhtml?token=74ace2fd-600b-473a-b654-baeb71908dd3

For manuscripts utilizing custom algorithms or software that are central to the research but not yet described in published literature, software must be made available to editors and reviewers. We strongly encourage code deposition in a community repository (e.g. GitHub). See the Nature Portfolio guidelines for submitting code & software for further information.

Data

Policy information about availability of data

All manuscripts must include a data availability statement. This statement should provide the following information, where applicable:

- Accession codes, unique identifiers, or web links for publicly available datasets
- A description of any restrictions on data availability
- For clinical datasets or third party data, please ensure that the statement adheres to our policy

Data and codes are available on a public repository, that will be made accessible upon publication. For the reviewer's and editor interest, they are available on a

provisory private link:
https://dataverse.cirad.fr/privateurl.xhtml?token=74ace2fd-600b-473a-b654-baeb71908dd3

Research involving human participants, their data, or biological material

Policy information about studies with human participants or human data. See also policy information about sex, gender (identity/presentation), and sexual orientation and race, ethnicity and racism.

Reporting on sex and gender	The research question concerns the oviposition behaviour of fruit flies, expressed only by females, thus only females were used in this manuscript.
Reporting on race, ethnicity, or other socially relevant groupings	n/a
Population characteristics	n/a
Recruitment	n/a
Ethics oversight	n/a

Note that full information on the approval of the study protocol must also be provided in the manuscript.

Field-specific reporting

Please select the one below that is the best fit for your research. If you are not sure, read the appropriate sections before making your selection.

☒ Life sciences ☐ Behavioural & social sciences ☐ Ecological, evolutionary & environmental sciences

For a reference copy of the document with all sections, see nature.com/documents/nr-reporting-summary-flat.pdf

Life sciences study design

All studies must disclose on these points even when the disclosure is negative.

Sample size	For Chemical analysis and EAG data, the recommended sample size is n=6 to 10 given low variability. We used n=7 for GC-MS sampling, and n=7 for EAG recorded in B. dorsalis. For all other tephritid species, this number was lower (usually n=4 or 5), which is balanced by the multiple conditions in which we observed a similar effect: 8 different species, 2 different doses, and 2 different simulation techniques (direct stimulation or GC-driven stimulation). Thus, the conclusion should not be held true for a given species, but rather as a general effect in this family of insects.<br>For behavioural data, we used an overly large number of samples (n=46 and n=60) because the experiment was replicated (see below)
Data exclusions	no data were excluded from the analysis
Replication	All attempts of replication were successful. In particular, the behavioural test confirmed an unconventional hypothesis that we formulated earlier : a preference that reverses as a function of dose. Surprised that the data matched our prediction, we decided to carry out the same experiment a second time, for confirmation. The two experiment have been combined into a single one in the manuscript.
Randomization	Fruit emissions were sampled at the maturity season of each fruit species - thus it could not have been random. Electrophysiological data were random, including the order of delivery of stimulations.
Blinding	Blinding was not relevant to our study.

Reporting for specific materials, systems and methods

We require information from authors about some types of materials, experimental systems and methods used in many studies. Here, indicate whether each material, system or method listed is relevant to your study. If you are not sure if a list item applies to your research, read the appropriate section before selecting a response.

Materials & experimental systems

n/a	Involved in the study
☒	☐ Antibodies
☒	☐ Eukaryotic cell lines
☒	☐ Palaeontology and archaeology
☐	☒ Animals and other organisms
☒	☐ Clinical data
☒	☐ Dual use research of concern
☐	☒ Plants

Methods

n/a	Involved in the study
☒	☐ ChIP-seq
☒	☐ Flow cytometry
☒	☐ MRI-based neuroimaging

Animals and other research organisms

Policy information about studies involving animals; ARRIVE guidelines recommended for reporting animal research, and Sex and Gender in Research

Laboratory animals	Female individuals of eight species of Tephritidae were used for the electrophysiological experiments: Bactrocera dorsalis (oriental fruit fly), Bactrocera zonata (peach fruit fly), Ceratitis capitata (medfly), Ceratitis catoirii (Mascarene fruit fly), Ceratitis quilicii (Cape fruit fly), Dacus demmerezi (Indian Ocean cucumber fruit fly), Neoceratitis cyanescens (tomato fruit fly) and Zeugodacus cucurbitae (melon fly). They originated from laboratory strains, which were reared 366 according to previously published procedures20,46. The individuals selected for electrophysiology had lived for respectively 12-15, 28-32, 10-14, 21-26, 13-17, 11-15, 10-16 and 25-31 days after emergence and belonged to the following generations: 62-64th, 179-180th, 107-108th, 311-312th, 77-79th, 59th, 64-369 65th and 7th.The behavioural experiments were conducted on female individuals of B. dorsalis from the 76-78th generations, 18-25 days after emergence.
Wild animals	The study did not involved wild animals
Reporting on sex	The finding applies only to females, since the studied behaviour (host search for oviposition) is female-specific.
Field-collected samples	Fruit emissions were collected on the tree, in the field, or after slicing the fruits immediately following harvesting.
Ethics oversight	No ethical guidance is required for studies involving insects.

Note that full information on the approval of the study protocol must also be provided in the manuscript.

Dual use research of concern

Policy information about dual use research of concern

Hazards

Could the accidental, deliberate or reckless misuse of agents or technologies generated in the work, or the application of information presented in the manuscript, pose a threat to:

No	Yes
☒	☐ Public health
☒	☐ National security
☒	☐ Crops and/or livestock
☒	☐ Ecosystems
☒	☐ Any other significant area

Experiments of concern

Does the work involve any of these experiments of concern:

No	Yes	
☒	☐	Demonstrate how to render a vaccine ineffective
☒	☐	Confer resistance to therapeutically useful antibiotics or antiviral agents
☒	☐	Enhance the virulence of a pathogen or render a nonpathogen virulent
☒	☐	Increase transmissibility of a pathogen
☒	☐	Alter the host range of a pathogen
☒	☐	Enable evasion of diagnostic/detection modalities
☒	☐	Enable the weaponization of a biological agent or toxin
☒	☐	Any other potentially harmful combination of experiments and agents

Plants

Seed stocks	n/a
Novel plant genotypes	n/a
Authentication	n/a
